# Role of percutaneous vertebroplasty with high-viscosity cement in the treatment of severe osteoporotic vertebral compression fractures

**DOI:** 10.1038/s41598-021-84314-6

**Published:** 2021-02-25

**Authors:** Kunpeng Li, Changbin Ji, Dawei Luo, Wen Zhang, Hongyong Feng, Keshi Yang, Hui Xu

**Affiliations:** 1 Department of Orthopaedics, Liaocheng People’s Hospital, No 67 Dongchang West Road, Liaocheng City, 252000 Shandong Province China; 2 Department of Ultrasonography, Liaocheng People’s Hospital, Liaocheng City, China

**Keywords:** Disability, Pain, Fracture repair, Quality of life

## Abstract

Severe osteoporotic vertebral compression fractures (OVCFs) were considered as relative or even absolute contraindication for vertebroplasty and kyphoplasty and these relevant reports are very limited. This study aimed to evaluate and compare the efficacy of vertebroplasty with high-viscosity cement and conventional kyphoplasty in managing severe OVCFs. 37 patients of severe OVCFs experiencing vertebroplasty or kyphoplasty were reviewed and divided into two groups, according to the procedural technique, 18 in high-viscosity cement percutaneous vertebroplasty (hPVP) group and 19 in conventional percutaneous kyphoplasty (cPKP) group. The operative time, and injected bone cement volume were recorded. Anterior vertebral height (AVH), Cobb angle and cement leakage were also evaluated in the radiograph. The rate of cement leakage was lower in hPVP group, compared with cPKP group (16.7% vs 47.4%, *P* = 0.046). The patients in cPKP group achieved more improvement in AVH and Cobb angle than those in hPVP group postoperatively (37.2 ± 7.9% vs 43.0 ± 8.9% for AVH, *P* = 0.044; 15.5 ± 4.7 vs 12.7 ± 3.3, for Cobb angle, *P* = 0.042). At one year postoperatively, there was difference observed in AVH between two groups (34.1 ± 7.4 vs 40.5 ± 8.7 for hPVP and cPKP groups, *P* = 0.021), but no difference was found in Cobb angle (16.6 ± 5.0 vs 13.8 ± 3.8, *P* = 0.068). Similar cement volume was injected in two groups (2.9 ± 0.5 ml vs 2.8 ± 0.6 ml, *P* = 0.511). However, the operative time was 37.8 ± 6.8 min in the hPVP group, which was shorter than that in the cPKP group (43.8 ± 8.2 min, *P* = 0.021). In conclusion, conventional PKP achieved better in restoring anterior vertebral height and improving kyphotic angle, but PVP with high-viscosity cement had lower rate of cement leakage and shorter operative time with similar volume of injected cement.

## Introduction

Osteoporotic vertebral compression fractures (OVCFs) are the most common fractures in patients with osteoporosis, which usually cause pain, deformities, and even mortality in elderly populations. The prevalence is increasing as population age, about 1.4 million new fractures every year^1,2^. Percutaneous vertebroplasty (PVP) has achieved satisfactory outcome in relieving pain and improving life quality for patients with OVCFs. However, severe OVCFs, the vertebral body collapsed to less than one-third of its original height, were regarded as contraindication for technical difficulties^3,4^. Therefore, these populations have to receive conservative treatment, but long-term bed rest may result in increased bone loss and secondary complications such as infection, bedsores, and muscles atrophy^5^.

Percutaneous kyphoplasty (PKP), a modified version of vertebroplasty, involves inflation of a balloon within the fractured vertebrae to create a cavity before bone cement injection. Several studies have reported that PKP could reduce the cement leakage rate, with equally better result in pain relief and life improvement, compared with PVP^6–8^. Recently, Baroud and his colleagues^9^ found in their experimental study that the leakage of bone cement reduced when the viscosity of the injected cement increased, and there was a strong relationship between them. Laboratory tests and clinical trials^10–12^ reported that the use of high-viscosity bone cement could reduce the leakage of bone cement in the PVP. However, reports that explicitly investigate the efficacy and safety of these modifications in severe OVCFs are limited.

This retrospective study presents the clinical and radiological outcomes of patients with severe OVCFs, who received PVP with high-viscosity cement and conventional PKP, in order to evaluate and compare efficacy and safety of conventional PKP and high-viscosity cement PVP in managing severe OVCFs.

## Method and materials

### Patient population

This retrospective study was conducted in Liaocheng People’s Hospital between Jan 2014 and Dec 2016, in which 37 patients of the severe OVCFs were reviewed. It was approved by the Ethics Committee of our Hospital and all patients provided written informed consents. All procedures in this study were performed according to the relevant guidelines and regulations.

In this study, all patients receiving PVP and PKP were reviewed. The inclusion criteria were as follow: 1. age above 65 years; 2. single-level OVCFs involving T10-L5 and absence of spinal cord or nerve compression symptoms; 3. fractured vertebrae collapsed to less than one third of its original height; 4. osteoporosis diagnosed by dual-energy X-ray absorptiometry, and bone mineral density (BMD) was less than 2.5 SD; 5. acute back pain associated with vertebral fracture (lasting less than 4 weeks) and visual analogue scale (VAS) ≥ 5; Patients were excluded if meeting to the following criteria: traumatic fracture, bone metastases, infectious diseases, tuberculosis, and a history of vertebroplasty and other thoracic or lumbar surgeries.

37 patients of severe OVCFs were divided into two groups, 18 patients who received the PVP with high-viscosity cement in the hPVP group and 19 patients who received the conventional PKP in the cPKP group.

### Procedural technique

The procedures were performed as described in previous reports^12,13^. Each patient was positioned prone on a radiolucent operating table. We adopted unilateral approach for all patients. 1% lidocaine was used to perform local infiltration anesthesia. An incision was made at the skin and then the needle was inserted to the junction of the anterior three quarters of the vertebral body carefully. The needle position was confirmed with C-arm fluoroscopy until it was satisfactory, then the needle was fixed in satisfactory position.

**Figure 1:**
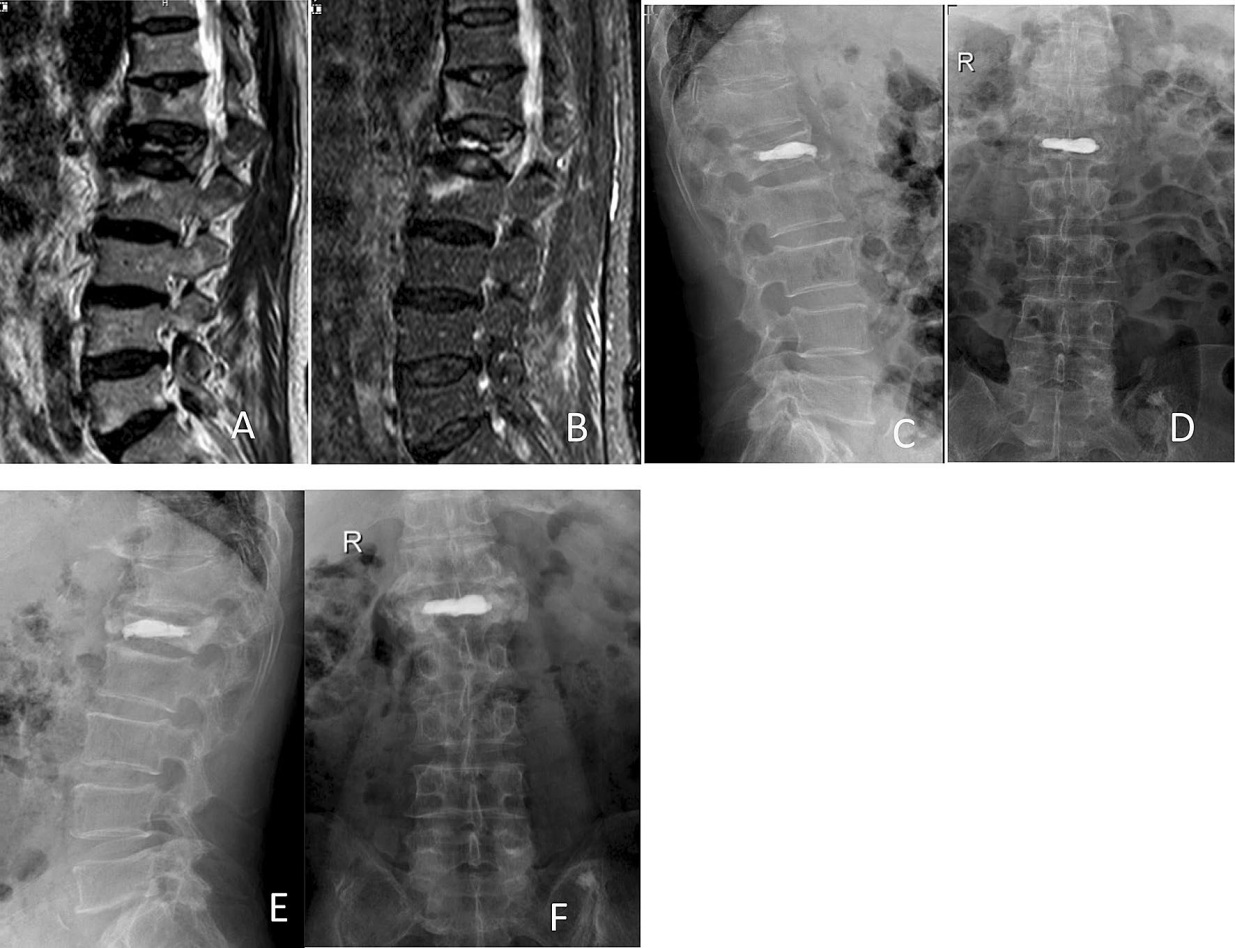
Preoperative and postoperative films of a 65-year-old woman with OVCFs of L1 vertebral body, treated with high-viscosity cement PVP. Preoperative sagittal MR image showing significant loss of the anterior vertebral body height at the L1 level (**A**), (**B**). Anterior–posterior and lateral films showing no leakage of bone cement postoperatively (**C**), (**D**) and at 1 year postoperatively (**E**), (**F**).

PVP: High-viscosity bone cement was adjusted to wiredrawing stage according to the instruction, and was then injected into vertebral body with special hydraulic propulsion pump (Fig. 1).

**Figure 2:**
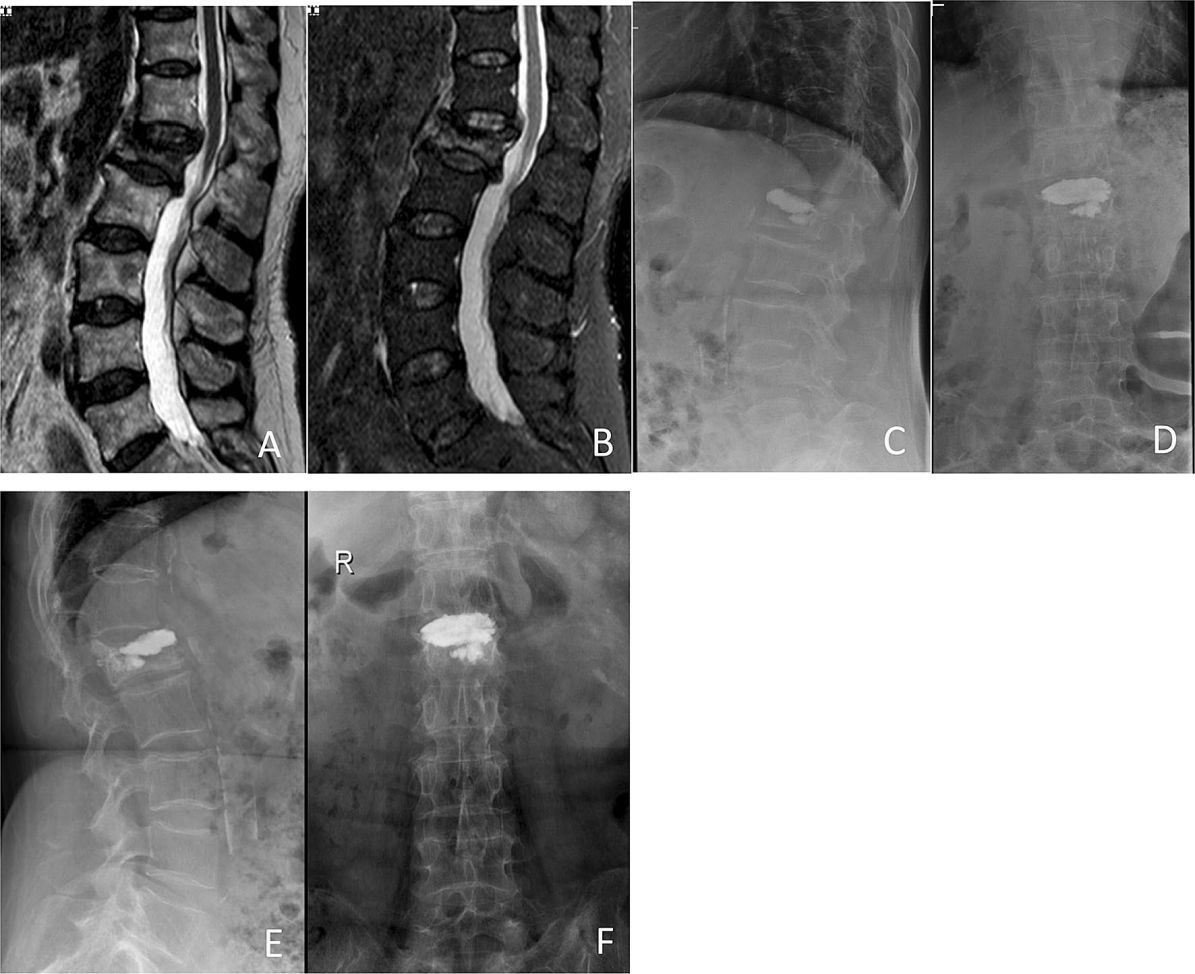
Preoperative and postoperative films of a 60-year-old woman with OVCFs of L1 vertebral body, treated with low-viscosity cement PKP. Preoperative MR image showing the compression fracture at the L1 level (**A**), (**B**). Anterior–posterior and lateral film showing the leakage of bone cement into the upper disc space postoperatively (**C**), (**D**) and at 1 year postoperatively (**E**), (**F**).

PKP: Balloon tamp was inserted through the previous needle channel and placed in the anterior three quarters of vertebrae body. The balloon was then inflated slowly until it contacted the endplate or the inflation pressure reached 200 psi. Conventional low-viscosity bone cement was manually injected to the cavity with a high pressure pump after removal of the balloon (Fig. 2).

Whenever the cement leakage occurred in epidural or paravertebral area or the bone cement reached the dorsal part of the vertebral body, the cement injection was stopped. The entire process was guided by C-arm fluoroscopy and bone cement spread out gradually.

### Postoperative managements

Patients were encouraged to start out-of-bed activities with lumbar brace after 24 h postoperatively. All patients received treatment with calcium and vitamin D supplements after surgery. Clinical and radiological assessments were performed for each patient in the outpatient clinic every three months since discharge from hospital.

### Observation index

Clinical outcome was measured with VAS score for back pain and Oswestry Disability Index (ODI) questionnaire. Operative time and volume of bone cement were also recorded.

Assessment of Cobb’s angle, anterior vertebral height (AVH) and bone cement leakage were based on preoperative and postoperative radiographs. Cobb’s angle was measured by the kyphotic angle from the superior endplate of the vertebral body one level above the fractured vertebra to the inferior endplate of the vertebral body one level below. AVH was measured and calculated according to previous report^14^. The location and severity of cement leakage were recorded. The severity of leakage was characterized as mild, moderate, or severe according to Georgy^15^. Additionally, the location was classified as the following: (1) the disk space, (2) the paravertebral areas, (3) the epidural space, and (4) the peripheral veins.

### Statistical analyses

Data analyses were performed with SPSS statistical package, Windows V17.0 (SPSS, Chicago, IL, U.S.A.). In this study, continuous data, including VAS, ODI, operative time, injected cement volume, AVH and Cobb angle were demonstrated as mean ± standard deviation and analyzed with two-sample t-test and ANOVA. Chi-squared test was used for categorical data analyses, such as the rate of cement leakage. For all analyses, a P value lower than 0.05 was considered as statistically significant.

## Result

In this series, 37 patients of severe OVCFs receiving PVP or PKP were included, with 18 in hPVP group and 19 in cPKP group. Patients' demographics and basic characteristics, including age, gender and fracture level were shown in the Table 1, and no difference was observed between two groups.

**Table 1:** Characteristics of the patient cohort in both groups.

Parameter	hPVP group	cPKP group	P value
Number of patients	18	19	
Age, years	73.9 ± 7.0 (66–87)	73.2 ± 6.2 (66–88)	0.758
Gender, males/females	4/14	6/13	0.535
Fracture level			0.858
Thoracic	8	9	
Lumbar	10	10	
Operative time (min)	37.8 ± 6.8	43.8 ± 8.2	0.021
Cement volume (ml)	2.9 ± 0.5	2.8 ± 0.6	0.511

### Clinical outcomes

The average VAS score for back pain in the hPVP group was 3.1 ± 0.9 at two days postoperatively, which was similar to that in the cPKP group (3.2 ± 0.6, *P* = 0.557). There was no significant difference in VAS score at one year after surgery between the two groups (1.3 ± 0.6 vs 1.5 ± 0.6, *P* = 0.416) (Table 2).

**Table 2:** The change of VAS for back pain in the pre-and post-operative period.

Group	preop	2d postop	1y postop
hPVP	6.9 ± 1.1	3.1 ± 0.9	1.3 ± 0.6
cPKP	7.1 ± 1.1	3.2 ± 0.6	1.5 ± 0.6
P value	0.559	0.557	0.416

Similar results were found in term of ODI scores at two days and one year after surgery (*P* = 0.637 and *P* = 0.822) (Table 3).

**Table 3:** The change of ODI (%) in the pre- and post-operative period.

Group	preop	2d postop	1y postop
hPVP	78.2 ± 7.8	25.9 ± 6.3	18.5 ± 5.6
cPKP	78.6 ± 7.9	27.0 ± 7.0	18.9 ± 4.4
P value	0.896	0.637	0.822

### Operative index

The operative time was 37.8 ± 6.8 min in the hPVP group, and 43.8 ± 8.2 min in the cPKP group. There was significant difference between the two groups (*P* = 0.021), suggesting that conventional PKP required longer time than PVP using high-viscosity bone cement. The injected bone cement volume was similar in two groups (2.9 ± 0.5 ml vs 2.8 ± 0.6 ml, *P* = 0.511) (Table 1).

### Radiological evaluation

The average AVH was 37.2 ± 7.9% in the hPVP, which was lower than cPKP group (43.0 ± 8.9%), and significant difference was found between the two groups (*P* = 0.044). At one year after surgery, there was also difference observed between two groups (34.1 ± 7.4 vs 40.5 ± 8.7 for hPVP and cPKP groups, *P* = 0.021) (Table 4).

**Table 4:** The change of AVH (%) in the pre- and post-operative period.

Group	preop	2d postop	1y postop
hPVP	22.9 ± 6.0	37.2 ± 7.9	34.1 ± 7.4
cPKP	22.1 ± 5.7	43.0 ± 8.9	40.5 ± 8.7
P value	0.687	0.044	0.021

Similar change occurred in Cobb’s angle. There was significant difference detected between the hPVP and cPKP groups postoperatively (15.5 ± 4.7 vs 12.7 ± 3.3, for hPVP and cPKP groups, *P* = 0.042). However, at one year postoperatively, no significant difference was found in term of Cobb’s angle between both groups (*P* = 0.068) (Table 5).

**Table 5:** The change of Cobb’s angle in the pre- and post-operative period.

Group	preop	2d postop	1y postop
hPVP	20.8 ± 5.8	15.5 ± 4.7	16.6 ± 5.0
cPKP	21.5 ± 5.6	12.7 ± 3.3	13.8 ± 3.8
P value	0.715	0.042	0.068

### Leakage rates and locations

Leakage rates and locations were presented in Table 6. The rate of cement leakage was 16.7% (3 of 18 patients) in the hPVP group, which was lower than 47.4% (9 of 19 patients) in the cPKP group. There was significant difference detected between two groups (*P* = 0.046). In the hPVP group, no patient had severe leakage (2 patients with mild leakage; one patient with moderate leakage). However, there are one patients of severe leakage, 3 moderate leakages, 5 mild leakages in the cPKP group.

**Table 6:** The location and grade of bone cement leakage in two groups.

Group	Leakage rate	Grade of leakage		
		Mild	Moderate	Severe
hPVP	3/18	2	1	0
cPKP	9/19	5	3	1
P value	0.046			

Group	Location of leakage			
	Disc space	Epidural space	Paravertebra	Peripheral vein
hPVP	2	0	1	0
cPKP	6	1	2	0

### Complications

No severe complications or postprocedural sequelae were encountered. There were two cases of new nonadjacent fracture in hPVP group and one case in cPKP group respectively, within one year postoperatively.

## Discussion

In the current study, we compare the efficacy and safety of conventional PKP and PVP with high-viscosity cement in the management of severe OVCFs. From the available data, the results showed that conventional PKP achieved better in the restoration of anterior vertebral height and kyphotic angle, but PVP with high-viscosity cement had lower rate of cement leakage and shorter operation time.

A great deal of clinical trials and systematic reviews^16–18^ had shown that PVP was a safe and effective choice for pain relief and quality of life improvement in the patients with OVCFs. However, severe OVCFs had been cited as relative or even absolute contraindication for technical difficulties to perform and resultant high risk of cement leakage. Several studies have reported that cement leakage in severe OVCFs, ranged from 43 to 45% detected by radiography to 78–91.9% detected by computed tomography, and was still the main risk of complication for conventional PVP^19–21^. Nevertheless, some authors adopted modifications of PVP or other methods to manage the patients of severe OVCFs, such as PKP, the use of high-viscosity bone cement in PVP and partial reduction before inserting the needle^21–23^. We retrospectively reviewed patients of severe OVCFs treated by conventional PKP or PVP with high-viscosity cement to compare the efficacy and safety of these two methods.

In this trial, both conventional PKP and PVP with high-viscosity cement gained satisfactory outcome in the patients of severe OVCFs. The results showed that the average ODI and VAS score decreased significantly after surgery in both groups. However, no difference was observed in terms of VAS and ODI between two groups, which indicated that both conventional PKP and high-viscosity PVP obtained equally better outcome in pain relief and life improvement. The similar effect of conventional PKP and PVP with high-viscosity cement may be attributed to the same mechanism of pain relief.

Major complications arising from PVP were closely associated with bone cement leakage^24,25^. Although most leakages were clinically asymptomatic, cement leakage was reported up to 91.9% in severe OVCFs and serious complications occurred in 3.9–7.5% of the patients who received vertebroplasty^4,21^. In our study, the rate of cement leakage was 16.7% in hPVP group, which was lower than 47.4% of cPKP group, indicating that PVP with high-viscosity cement resulted in lower rate of leakage compared with low-viscosity cement PKP in severe OVCFs. Habib^10^ reported that the filling uniformity increased and cement leakage reduced in all high-viscosity cement compared to the low-viscosity cement and he attributed the decrease of cement leakage in vertebral augmentation procedures to the uniform cement filling of high-viscosity system. We concluded that high-viscosity cement could reach maximum viscosity at a quicker rate and remain at this higher viscosity for a longer duration, which allows for a longer injection time and therefore easier injection.

Zhu et al.^26^ previously reported that the volume of injected bone cement was determined to be a strongly associated factor with bone cement leakage, and the volume of cement in vertebroplasty was related to cement viscosity. This was not consistent with our results. In our study, similar volume of bone cement was injected in two groups (2.9 ± 0.5 ml vs 2.8 ± 0.6 ml), but less cement leakage occurred in patients receiving the high-viscosity cement PVP. This may indicate that lower incidence of cement leakage in hPVP group was not due to the injection volume, but the high viscosity of injected cement.

The restoration of vertebral body height and kyphotic deformity was very important, especially for patients of severe OVCFs. A previous study reported that the PVP with the high-viscosity bone cement and PKP could restore the anterior vertebrae height and improve the kyphotic angle in the patients of severe OVCFs^21^. Our radiologic results showed that the initial correction of AVH in the cPKP group increased to 43.0 ± 8.9 from 22.1 ± 5.7, and to 37.2 ± 7.9 from 22.9 ± 6.0 in the hPVP group, and Cobb’s angle decreased to 12.7 ± 3.3 from 21.5 ± 5.6 in the cPKP group, and to 15.5 ± 4.7 from 20.8 ± 5.8 in the hPVP group respectively, which suggested that both the AVH and Cobb had been significantly improved in two groups after surgery. These were consistent with the previous reports. However, conventional PKP achieved greater correction of anterior vertebral height and kyphotic angle compared to high-viscosity PVP in the severe OVCFs. At the last follow-up, there was significant difference in AVH between two groups, but no difference in Cobb’s angle.

In this series, the operative time in the hPVP group was shorter than that of the cPKP group, suggesting that the use of high viscosity cement enabled the procedure of PVP to complete more easily. Georgy^15^ believed that high-viscosity cement may alleviate the need for bone tamps and cavity creation within the vertebral body, thus significantly reducing the number of steps and procedure time. Furthermore, no inflation of a balloon within a collapsed vertebral body also reduced the duration of this procedure.

There are several limitations to this study. First, the chief limitation was small number of patients enrolled in this trial, which may impair the ability to assess the effect of high viscosity cement vertebroplasty on management of severe OVCFs when compared to low viscosity kyphoplasty. Second, this study was a retrospective trial at a single center, not prospective, blinded and randomized. In addition, it only focused on short-term results (within one year), and findings may not be indicative of long-term results. In the future, prospective randomized controlled studies, enrolling more patients and a long-time follow-up period, will be necessary to properly evaluate the efficacy and safety of the high-viscosity in severe OVCFs.

## Conclusion

The results of this study confirmed that high-viscosity PVP and conventional PKP were both safe and effective treatments in improving the quality of life and relieving pain for patients of severe OVCFs. Based on these available data, conventional PKP achieved better in restoring anterior vertebral height and improving kyphotic angle, but high-viscosity PVP had lower rate of cement leakage and shorter operative time with similar volume of injected cement.
